# Common-mode noise cancellation circuit for wearable ECG

**DOI:** 10.1049/htl.2016.0083

**Published:** 2017-04-20

**Authors:** Mutsumi Noro, Daisuke Anzai, Jianqing Wang

**Affiliations:** Nagoya Institute of Technology, Nagoya 466-8555, Japan

**Keywords:** electrocardiography, medical signal processing, signal denoising, diseases, patient monitoring, biomedical electrodes, photoresistors, cadmium compounds, biomedical electronics, common-mode noise cancellation, wearable ECG, electrocardiogram, heart disease monitoring, healthcare, medical applications, contact resistances, sensing electrodes, external electromagnetic field, ECG detection circuit, differential mode interference voltage, cadmium sulphide photoresistors, CdS

## Abstract

Wearable electrocardiogram (ECG) is attracting much attention for monitoring heart diseases in healthcare and medical applications. However, an imbalance usually exists between the contact resistances of sensing electrodes, so that a common mode noise caused by external electromagnetic field can be converted into the ECG detection circuit as a differential mode interference voltage. In this study, after explaining the mechanism of how the common mode noise is converted to a differential mode interference voltage, the authors propose a circuit with cadmium sulphide photo-resistors for cancelling the imbalance between the contact resistances and confirm its validity by simulation experiment. As a result, the authors found that the interference voltage generated at the wearable ECG can be effectively reduced to a sufficient small level.

## 1 Introduction

In the near future, it is desirable to use wearable sensors to automatically collect various vital data such as blood pressure, body temperature, electrocardiogram (ECG), electroencephalogram and so on, in daily life. Especially, in an aging society, heart disease is one of the major problems. To enable early detection of a heart disease, daily examination is a must. Therefore, the wearable sensors need to have a communication function to send the acquired vital data to a coordinator on the human body, and the coordinator then forwards the collected vital data to a hospital or medical centre for healthcare administration [1–3]. ECG signal is typically detected by pasting a plurality of electrodes directly to the skin. However, due to the contact condition of the electrodes each other, an imbalance usually exists between the contact resistances, which will lead a common mode noise caused by external electromagnetic field be superimposed on the ECG detection circuit as an interference voltage [4, 5]. If the frequency of external electromagnetic field is known in advance, it is possible to remove the interference voltage by using a filter. If it is not known in advance, the interference voltage cannot be easily removed by using a filter. Therefore, in this study, we propose a novel circuit structure for detecting and cancelling the imbalance between contact resistances of the ECG sensing electrodes, and show its effectiveness in reducing the common mode noises from external electromagnetic fields.

## 2 Mechanism of generation of interference voltage

**Fig. 1:**
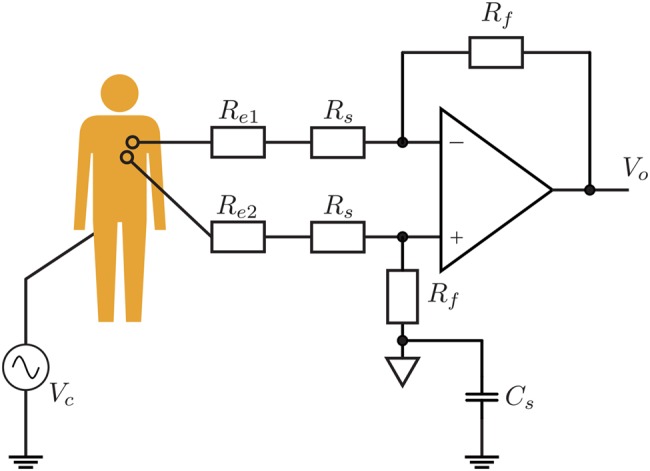
Common mode equivalent circuit of the ECG signal detection circuit

Let us consider that a ECG signal is obtained by two electrodes pasted directly to the human body and amplified using a differential amplifier. When an external electromagnetic field is irradiated on the human body standing on the earth, a common mode noise voltage will be generated between the human body and the earth ground. Fig. 1 shows the common mode equivalent circuit of the ECG signal detection circuit. If we denote $R_{e1}$, $R_{e2}$ as the contact resistances between each electrode and the human body, respectively, $R_s$, $R_f$ as the resistances for the differential amplifier, and $C_s$ as the parasitic capacitance between the circuit ground and the earth ground, the output interference voltage $V_{\rm o}$ of the differential amplifier circuit can be expressed by the following equation:

(1) $$V_{\rm o} = \displaystyle{{\,j\omega C_sR_f\lpar R_{e1} - R_{e2}\rpar } \over {R_A + R_B + j\omega C_sR_A\lpar R_B + R_f\rpar }}V_c\eqno\lpar 1\rpar $$

where $R_A = \lpar R_{e1} + R_s\rpar $, $R_B = \lpar R_{e2} + R_s\rpar $. From (1), if the contact resistances between the two electrodes and the human body are imbalanced, i.e. $R_{e1}$ is not equal $R_{e2}$, the interference voltage $V_{\rm o}$ at the output of the differential amplifier circuit will be superimposed on the ECG signal. For example, let us consider a 80 kHz wireless power transfer system being used for automobile. Under the assumption that the contact resistor $R_{e1}$ and $R_{e2}$ equal to 7 and 13 kΩ respectively, and the resistor $R_s$ and $R_f$ in the differential amplifier circuit are 10 kΩ and 10 MΩ, respectively, the ratio of denominator to numerator of (1) will be only 1/5. It means that a differential voltage of 0.2 V will be added to the ECG detector output if the common mode voltage is 1 V. Hence, a significant interference voltage can appear at the output of the differential amplifier especially for higher frequency interferences. While when the contact resistances value of $R_{e1}$ and $R_{e2}$ are the same (that is, there is no imbalance between the contact resistances), the common mode noise $V_c$ will not be converted to a differential mode noise, and the interference voltage $V_{\rm o}$ will be zero. Therefore, making the contact resistances of the two sensing electrodes at a balanced condition can avoid the common mode noise to be superimposed on the ECG signal.

## 3 Configuration of the contact resistance imbalance cancellation circuit

**Fig. 2:**
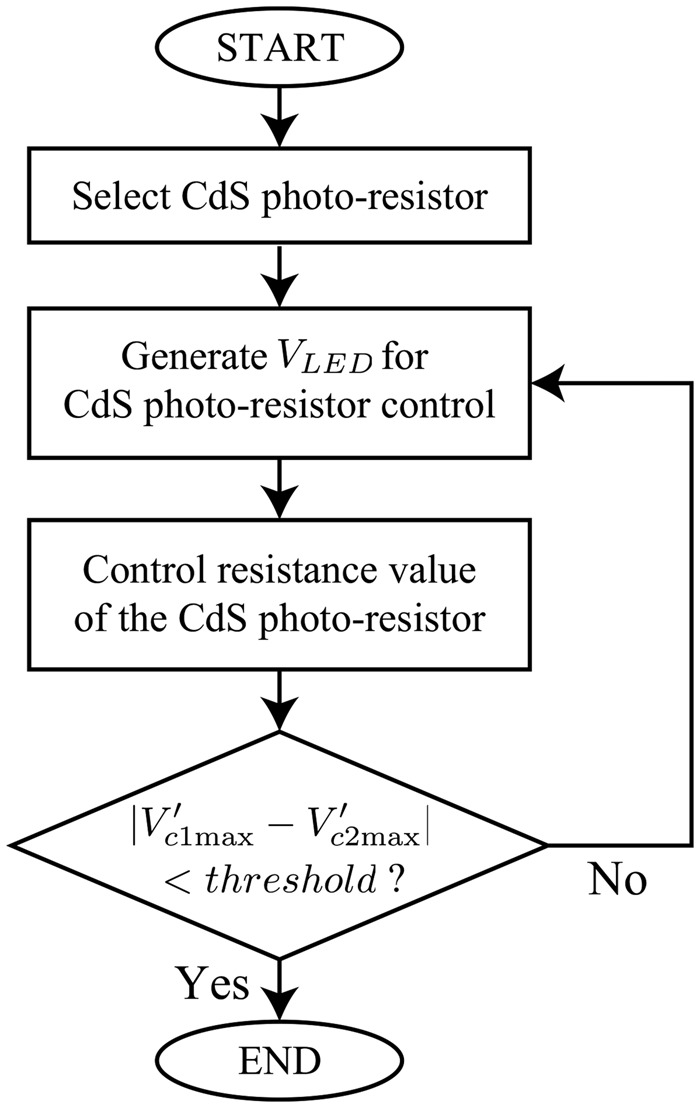
Flowchart of the proposed common mode noise cancellation method

**Fig. 3:**
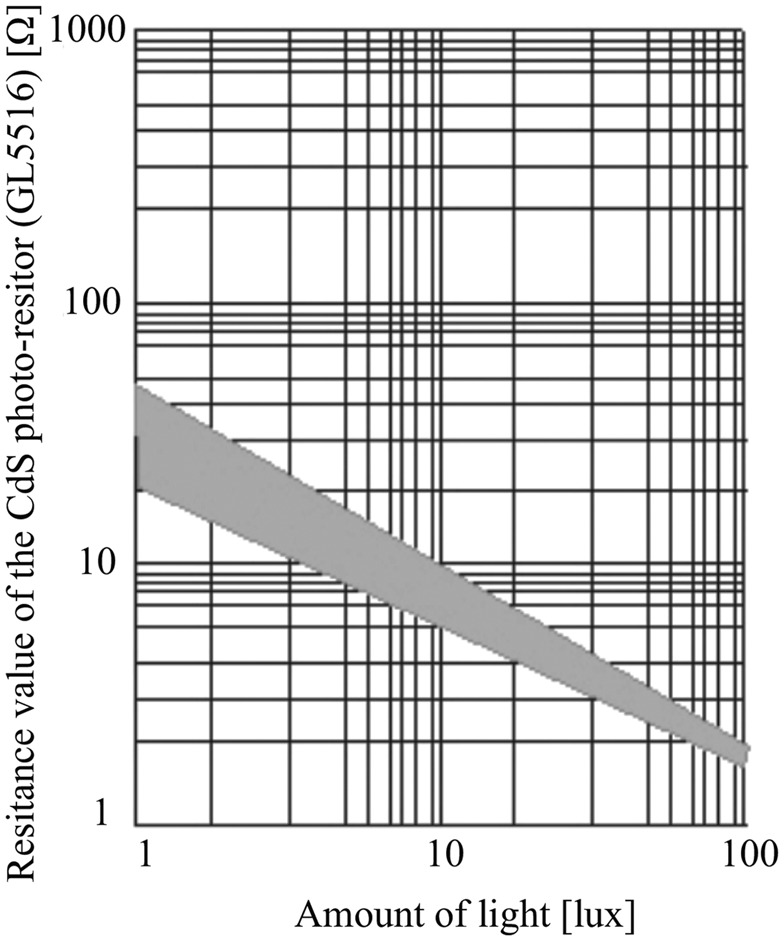
Illuminance resistance characteristics of CdS

**Fig. 4:**
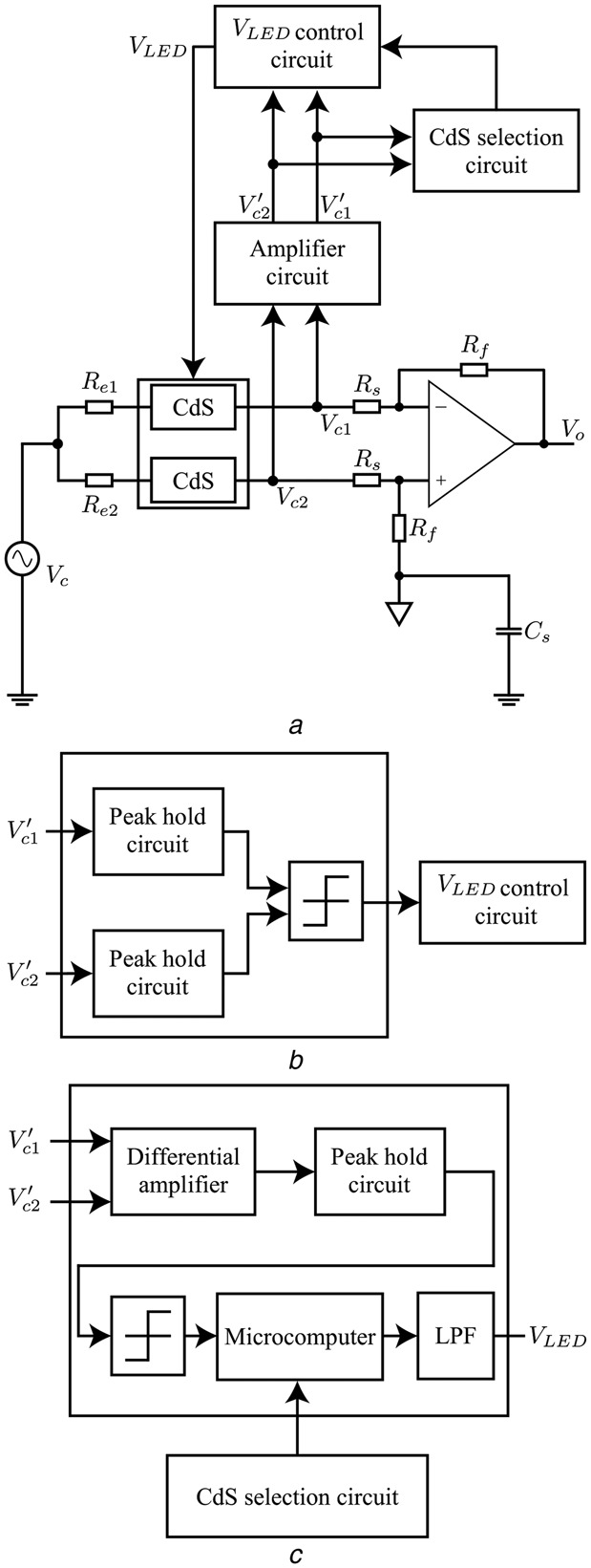
Block diagram of imbalance cancellation circuit of contact resistances *a* Main circuit *b* Cds selection circuit *c V*_LED_ control circuit

Fig. 2 shows the flowchart of the proposed common mode noise cancellation method. First, we detect the imbalance of the contact resistances between the ECG signal detection electrodes. Then, in order to compensate for the imbalance between the resistances, two variable resistors are inserted between the electrodes and the inputs of the differential amplifier circuit, respectively. The resistance value of each variable resistor is controlled according to the magnitude of the voltage applied to the variable resistor. For the variable resistors, we adopted cadmium sulphide (CdS). A CdS photo-resistor is light-controlled. When the light enters the device, the resistance of the CdS reduces. We therefore also adopt a light emitting diode (LED) to control the CdS photo-resistor. Fig. 3 shows the illuminance resistance characteristics of CdS photo-resistor [6]. Moreover, Fig. 4 shows the block diagram of the imbalance cancellation circuit of the contact resistance with the CdS photo-resistor. $V_{c1}$ and $V_{c2}$ in Fig. 4 are mainly from the common mode noise component, because the ECG signal is assumed much smaller than the common mode noise, as described in Fig. 6 of [4]. To equalise the contact resistances between the electrodes for ECG detection circuit, it is necessary to control either one of the CdS photo-resistors. As shown in Fig. 4*b*, first, to select which CdS photo-resistor to control, their initial resistance values are set as the minimum value. Because the magnitudes of the common mode voltage $V_{c1}$ and $V_{c2}$ depend on the external electromagnetic fields, they are first amplified to a level that can be compared by the comparator circuit as necessary. Then, the peak hold circuits detect the maximum values, and the comparator circuit compares the maximum values of ${V}^{\prime}_{c1}$ and ${V}^{\prime}_{c2}$. The CdS photo-resistor with larger voltage value is selected for control. In addition, the time interval for re-selecting the CdS photo-resistors is chosen in advance taking the usage environment into account. After determining which CdS photo-resistor to use by the CdS selection circuit, the resistance value of the CdS photo-resistor is controlled by applying the output voltage of the $V_{{\rm LED}}$ control circuit to the LED. As shown in Fig. 4*c*, first, to take the difference in the differential amplifier output, we amplify the common mode voltage ${V}^{\prime}_{c1}$ and ${V}^{\prime}_{c2}$. Then, the differential amplifier detects the voltage difference between ${V}^{\prime}_{c1}$ and ${V}^{\prime}_{c2}$, and the peak hold circuit detects the maximum value of the voltage difference. Furthermore, through the comparator circuit, the voltage difference is compared to a predetermined threshold value. If it is larger than the threshold value, the comparator circuit outputs High, if it is smaller, the comparator circuit outputs low. When the output of comparator circuit is high, the microcomputer raises one counter and outputs a pulse width modulation signal corresponding to the value of the counter. Subsequently, through a low pass filter (LPF) the signal is converted into a DC voltage, and it controls the voltage $V_{{\rm LED}}$ applying to the LED. The LPF is a first order low pass RC filter with a resistor of 100 Ω and a capacitor of 470 μF. Its cut-off frequency is 3.4 Hz, and the stop-band loss is about 40 dB. The above-described process is repeated at a constant period. The period is determined from the lowest frequency of the common mode noises in the usage environment. The higher the frequency is, the shorter the period should be. When the two contact resistances between the electrodes and the human body are nearly balanced, the voltage difference between $V_{c1}$ and $V_{c2}$ will be sufficiently small. At this time, the output voltage from the peak hold circuit falls below the threshold value, and the comparator circuit outputs Low. Then the count of the microcomputer will not change, and $V_{{\rm LED}}$ is fixed. This makes us possible to remove the interference voltage caused by the common mode noise appearing at the ECG detection circuit.

**Fig. 5:**
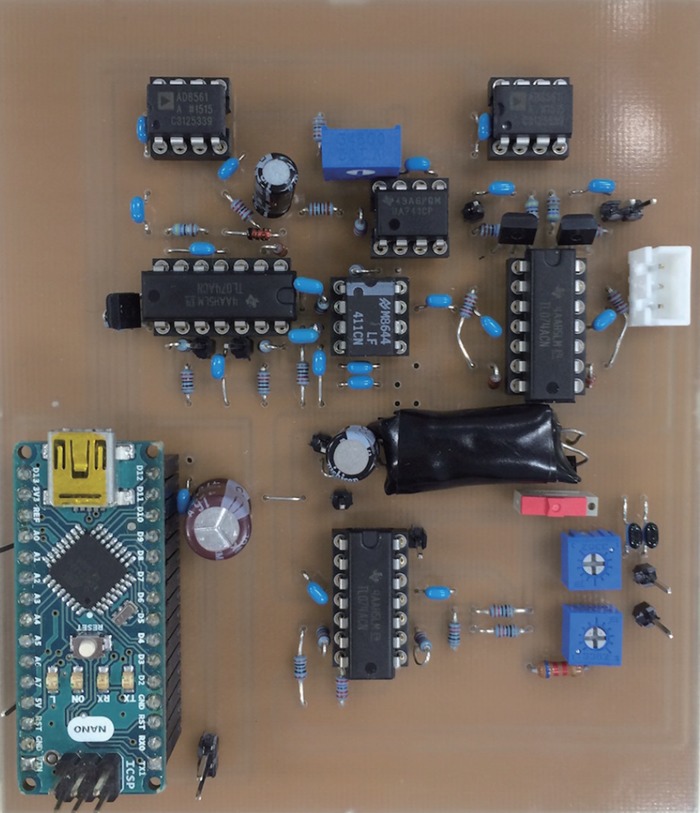
View of circuit for experimental validation

**Fig. 6:**
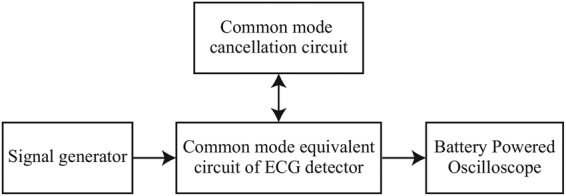
Block diagram of the simulation experiment environment

## 4 Validation of reduction effect of common mode noise

**Fig. 7:**
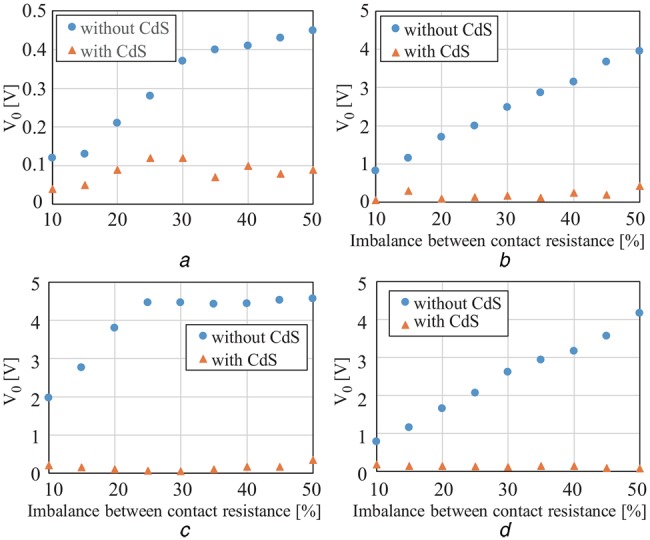
Comparison of interference voltage $V_o$ against imbalance between contact resistances in the cases of with and without the proposed cancellation circuit *a* Frequency: 60 Hz *b* Frequency: 1 kHz *c* Frequency: 10 kHz *d* Frequency: 80 kHz

**Fig. 8:**
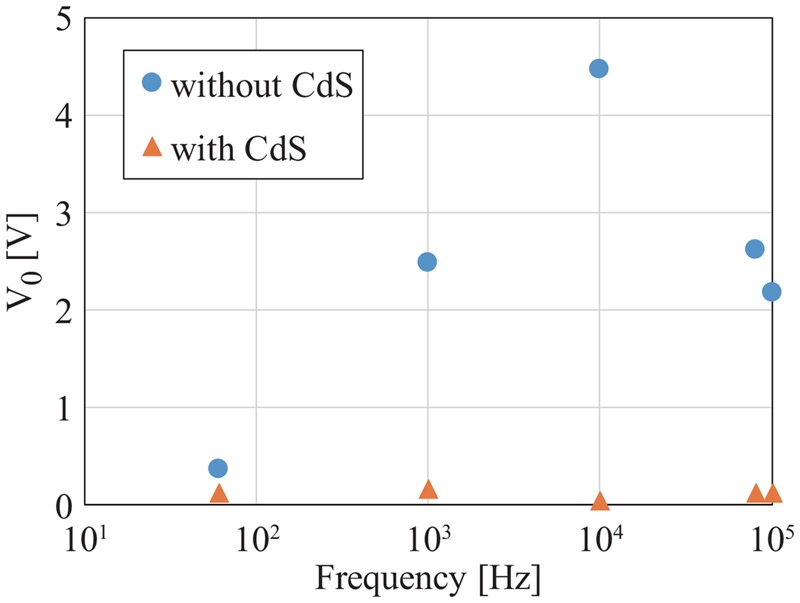
Interference voltage $V_o$ against frequency at an imbalance of contact resistances of 30%

To verify the effectiveness of the proposed circuit, we produced the circuit proposed in Fig. 4, and conducted a simulation experiment. Fig. 5 shows the produced circuit, and Fig. 6 shows the block diagram of the experiment environment. A signal generator was used to generate the common mode noise voltage, and the differential mode voltage $V_{\rm o}$ was measured with a digital oscilloscope to observe its reduced effect. In the circuit of Fig. 4*a*, the contact resistance values $R_{e1}$ and $R_{e2}$ between the electrodes and the human body were determined by a percentage of imbalance from 10 kΩ; for example, if the percentage is 10%, the resistance values of $R_{e1}$ and $R_{e2}$ are 9 and 11 kΩ, respectively. The gain of the differential amplifier was set to 60 dB. The voltage amplitude of $V_c$ was set to 1 V, and the frequency of common mode noise was changed from 60 Hz to 100 kHz. The parasitic capacitance Cs between the circuit ground and the earth ground was assumed as 200 pF [7]. Table 1 summarises the models and values of the circuit for validation experiment. The consumption power of the validation circuit is nearly 500 mW. It should be noted that, however, most of the powers are consumed in the microcomputer and the LED lighting part. If we can replace these parts with an integrated circuit specially designed for our use, the consumption power can be reduced largely. Fig. 7 shows the experimental results with and without the proposed circuit at several specified frequencies of 60 Hz, 1 kHz, 10 kHz and 80 kHz. The first frequency is being used as a commercial power frequency, and the last frequency is being used for wireless power charging for cars. Fig. 8 shows the result at the imbalance of 30%. From these results, we confirmed significant reduction of the interference voltage superimposed on the differential amplifier output. For example, when the imbalance was 30% and the frequency was 10 kHz, the interference voltage was decreased to about 1/9. On the other hand, it was observed from Fig. 8 that the interference voltage $V_{\rm o}$ of the differential amplifier attenuated at higher frequencies in the case without the cancellation circuit. This was because of its own characteristic of the operational amplifier. With the aid of the proposed cancellation circuit, the interference voltage $V_{\rm o}$ was reduced to a sufficiently small level. Incidentally, since $V_{{\rm LED}}$ only use discrete values, the interference voltage $V_{\rm o}$ did not converge to a constant value. This is the reason that there are some discontinuous points in Fig. 7, and they can be improved by mincing the control voltage $V_{{\rm LED}}$.

**Table 1:** Various parameters in the simulation experiment and the various model number

amplitude of the common mode noise source	1 V
frequency of common mode noise source	60 Hz–100 kHz
gain of the differential amplifier in the detecting circuit	60 dB
parasitic capacitance	200 pF
operational amplifier of the differential amplifier and the peak hold circuit	TL074ACN (Texas instruments)
comparator	AN8561 (analogue devices)
microcomputer	arduino nano (arduino)

## 5 Conclusion

When we detect the ECG signal, a common mode noise caused by external electromagnetic field may be superimposed on the ECG detection circuit as an interference voltage. To remove the common mode noise, the mechanism of generation of the interference voltage has been revealed as a conversion from the common mode voltage, caused by external electromagnetic field, to the differential mode voltage due to an imbalance between the contact resistances of the ECG sensing electrodes. Then, in order to equalise the contact resistances, we have proposed a cancellation circuit of contact resistance imbalance by using of CdS photo-resistors as variable resistors. After that, we have produced the circuit, and examined the effectiveness of the proposed circuit through a simulation experiment. As a result, we have showed that the proposed circuit can significantly reduce the interference voltage at the ECG detection circuit to a sufficiently small level. The future subject is to embed the proposed cancellation circuit into actual wearable ECG.
